# Supplementary material for: Evaluating prediction of short‐term tolerability of five type 2 diabetes drug classes using routine clinical features: UK population‐based study
Source: Diabetes Obes Metab. 2025 May 16;27(8):4320–9. doi: 10.1111/dom.16470 (PMC12232322; doi:10.1111/dom.16470)
Supplement: Supplementary file 1 — Data S1. Supporting information. [file DOM-27-4320-s001.pdf]

Supplementary material for:

Evaluating prediction of short-term tolerability of five type 2 diabetes drug classes using routine clinical features: UK population-based study

Pedro Cardoso, Katie G. Young, Rhian Hopkins, Bilal A. Mateen, Ewan R. Pearson, Andrew T. Hattersley, Trevelyan J. McKinley, Beverley M. Shields, John M. Dennis, on behalf of the MASTERMIND consortium

Supplementary Materials:

Tables:

sTable 1: Therapy discontinuation at 3-months (primary outcome), 6-months, and 12-months. N's represent the number of drug initiations included in the analysis of 3-month discontinuation. Breakdown of patient numbers for each therapy at each time point. Data are number (%).

Therapy	GLP-1RA (n=16,347)	DPP4i (n=71,460)	SGLT2i (n=50,510)	TZD (n=5,081)	SU (n=38,796)
3-month discontinuation					
No	13,995 (85.6)	62,296 (87.2)	42,961 (85.1)	4,087 (80.4)	32,717 (84.3)
Yes	2,352 (14.4)	9,164 (12.8)	7,549 (14.9)	994 (19.6)	6,079 (15.7)
6-month discontinuation					
No	12,464 (76.2)	56,958 (79.7)	38,761 (76.7)	3,604 (70.9)	29,694 (76.5)
Yes	3,173 (19.4)	12,306 (17.2)	9,850 (19.5)	1,332 (26.2)	7,904 (20.4)
Missing	710 (4.3)	2,196 (3.1)	1,899 (3.8)	145 (2.9)	1,198 (3.1)
12-month discontinuation					
No	9,387 (57.4)	46,957 (65.7)	30,467 (60.3)	2,836 (55.8)	24,770 (63.8)
Yes	4,256 (26.0)	16,589 (23.2)	12,534 (24.8)	1,768 (34.8)	10,190 (26.3)
Missing	2,704 (16.5)	7,914 (11.1)	7,509 (14.9)	477 (9.4)	3,836 (9.9)

sTable 2: Breakdown of patient numbers across subgroups of candidate predictors in Figure 2. Data are number (%).

Therapy	GLP-1RA (n=16,347)	DPP4i (n=71,460)	SGLT2i (n=50,510)	TZD (n=5,081)	SU (n=38,796)
Current age, years					
<50	3,361 (20.6)	9,906 (13.9)	9,979 (19.8)	883 (17.4)	7,387 (19.0)
50-59	5,555 (34.0)	17,103 (23.9)	17,483 (34.6)	1,480 (29.1)	10,525 (27.1)
60-69	4,879 (29.8)	19,792 (27.7)	15,739 (31.2)	1,439 (28.3)	10,687 (27.5)
70+	2,552 (15.6)	24,659 (34.5)	7,309 (14.5)	1,279 (25.2)	10,197 (26.3)
Duration of diabetes, years					
<3	2,014 (12.3)	12,317 (17.2)	7,347 (14.5)	753 (14.8)	10,908 (28.1)
3-5	3,237 (19.8)	15,558 (21.8)	10,585 (21.0)	1,176 (23.1)	10,583 (27.3)
6-9	4,361 (26.7)	18,210 (25.5)	13,322 (26.4)	1,535 (30.2)	9,765 (25.2)
10+	6,735 (41.2)	25,375 (35.5)	19,256 (38.1)	1,617 (31.8)	7,540 (19.4)
Biomarkers					
BMI, kg/m^2					
<30	1,861 (11.4)	28,533 (39.9)	13,994 (27.7)	2,138 (42.1)	15,139 (39.0)
30-35	4,837 (29.6)	20,967 (29.3)	16,033 (31.7)	1,443 (28.4)	11,233 (29.0)
35+	9,217 (56.4)	18,588 (26.0)	18,776 (37.2)	1,315 (25.9)	10,168 (26.2)
Missing	432 (2.6)	3,372 (4.7)	1,707 (3.4)	185 (3.6)	2,256 (5.8)
HbA_{1c}, mmol/mol*					
53-64	2,516 (15.4)	21,314 (29.8)	10,009 (19.8)	997 (19.6)	8,536 (22.0)
64-75	4,782 (29.3)	23,851 (33.4)	15,602 (30.9)	1,777 (35.0)	11,122 (28.7)
75-86	3,996 (24.4)	12,831 (18.0)	11,209 (22.2)	1,092 (21.5)	7,173 (18.5)
86+	5,053 (30.9)	13,464 (18.8)	13,690 (27.1)	1,215 (23.9)	11,965 (30.8)
eGFR, ml/min per 1.73m^2					
<75	3,562 (21.8)	21,751 (30.4)	6,175 (12.2)	1,206 (23.7)	9,083 (23.4)
75-90	3,067 (18.8)	15,116 (21.2)	10,953 (21.7)	1,028 (20.2)	7,834 (20.2)
90+	9,698 (59.3)	34,481 (48.3)	33,300 (65.9)	2,842 (55.9)	21,789 (56.2)
Missing	20 (0.1)	112 (0.2)	82 (0.2)	5 (0.1)	90 (0.2)

sTable 3: Discrimination values (AUC) for discontinuation BART models at 3-months, 6-months and 12-months. Models were fitted to all therapies simultaneously.

Model	3-month discontinuation	6-month discontinuation	12-month discontinuation
Overall	0.612 (0.608; 0.616)	0.611 (0.608; 0.615)	0.614 (0.610; 0.617)
GLP-1RA	0.610 (0.596; 0.623)	0.594 (0.583; 0.606)	0.585 (0.574; 0.596)
DPP4i	0.609 (0.602; 0.616)	0.607 (0.601; 0.613)	0.613 (0.607; 0.618)
SGLT2i	0.608 (0.600; 0.615)	0.611 (0.605; 0.618)	0.614 (0.608; 0.620)
TZD	0.596 (0.575; 0.617)	0.612 (0.593; 0.631)	0.607 (0.590; 0.625)
SU	0.605 (0.597; 0.614)	0.603 (0.595; 0.610)	0.605 (0.598; 0.612)

Figures:

sFig. 1: CPRD patient flow and inclusion criteria for the analysis cohorts. Baseline HbA_{1c} is defined as the closest HbA_{1c} to drug initiation in the previous 6-months. Other biomarkers were defined as the closest measure to drug initiation in the previous 2 years.

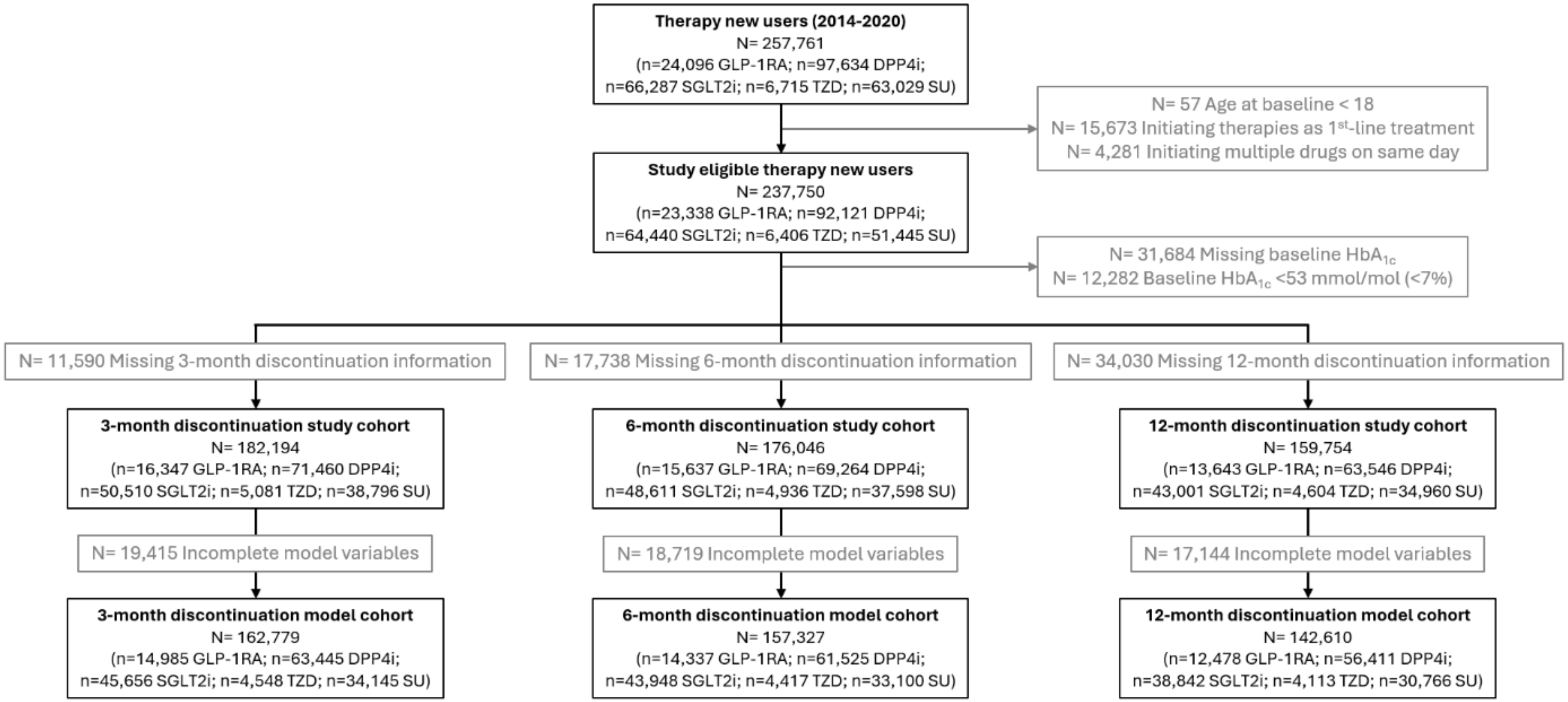

sFig. 2: Proportion of individuals with T2D discontinuing the five drug classes at 3-, 6- and 12-months, stratified by sociodemographic and clinical features.

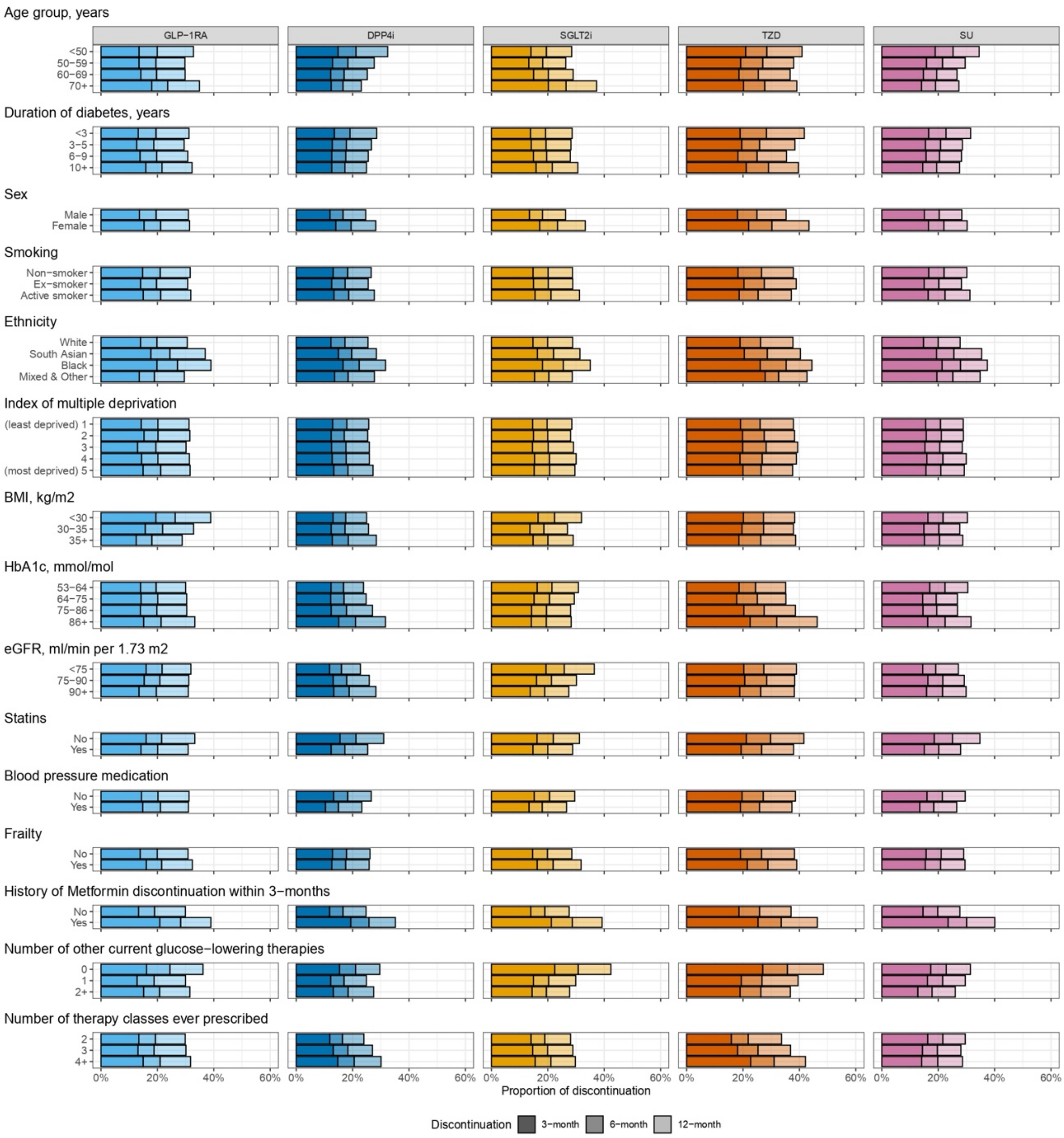

sFig. 3: Calibration plots for discontinuation BART models at 3-months (A), 6-months (B) and 12-months (C), for overall (1) and per therapy (2) discontinuation. (A) discontinuation at 3-months. (B) discontinuation at 6-months. (C) discontinuation at 12-months. (1) overall discontinuation. (2) per therapy discontinuation. Calibration plot shows the predicted and observed discontinuation for groups defined by decile of predicted discontinuation risk. Black line represents perfect calibration.

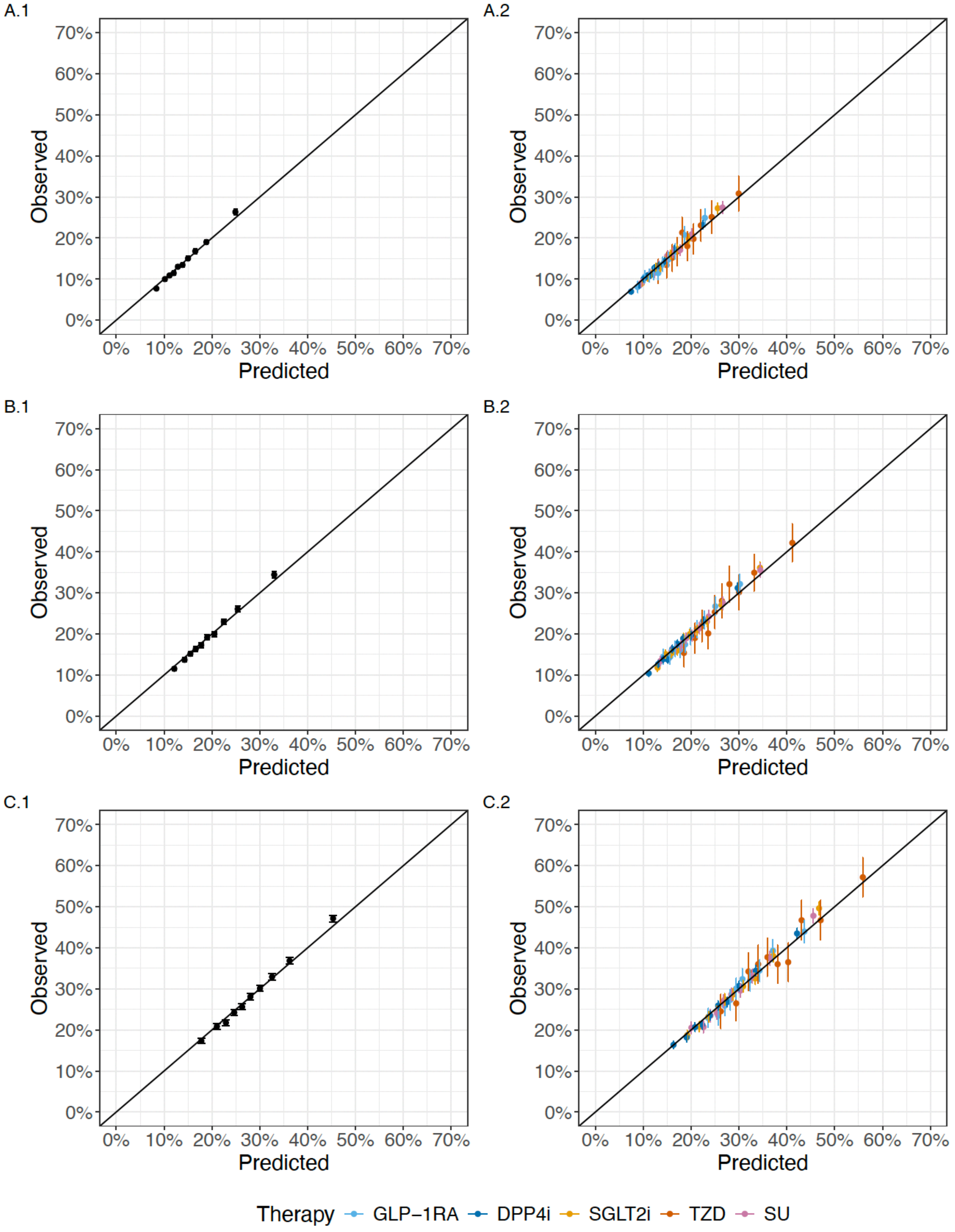

sFig. 4: Precision-recall curve plot for 3-month discontinuation. The line represents the precision-recall curve for the mean predicted probability of discontinuation. The different thresholds for defining positive cases are represented by the colour scale. The plot help understand the model's ability to find positive cases and compare the model's ability to not label a negative case as positive (precision) and the model's ability to find all positive cases (recall). The higher the area under the curve, the better the model (similar to AUROC).

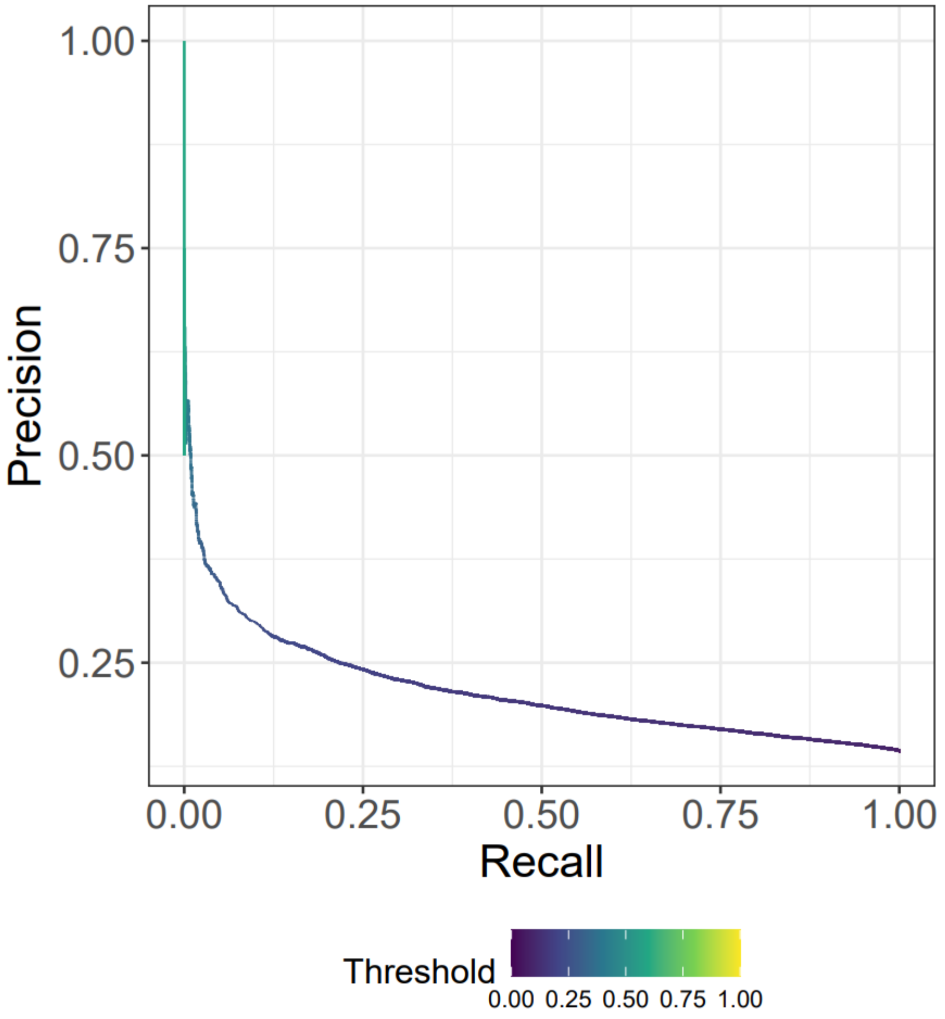

sFig. 5: Discrimination plots for discontinuation BART models at 3-months (A), 6-months (B) and 12-months (C), for overall (1) and per therapy (2) discontinuation. (A) discontinuation at 3-months. (B) discontinuation at 6-months. (C) discontinuation at 12-months. (1) overall discontinuation. (2) per therapy discontinuation. AUROC = aura under the receiver operating characteristics curve. A breakdown of AUC values can be found in sTable 3.

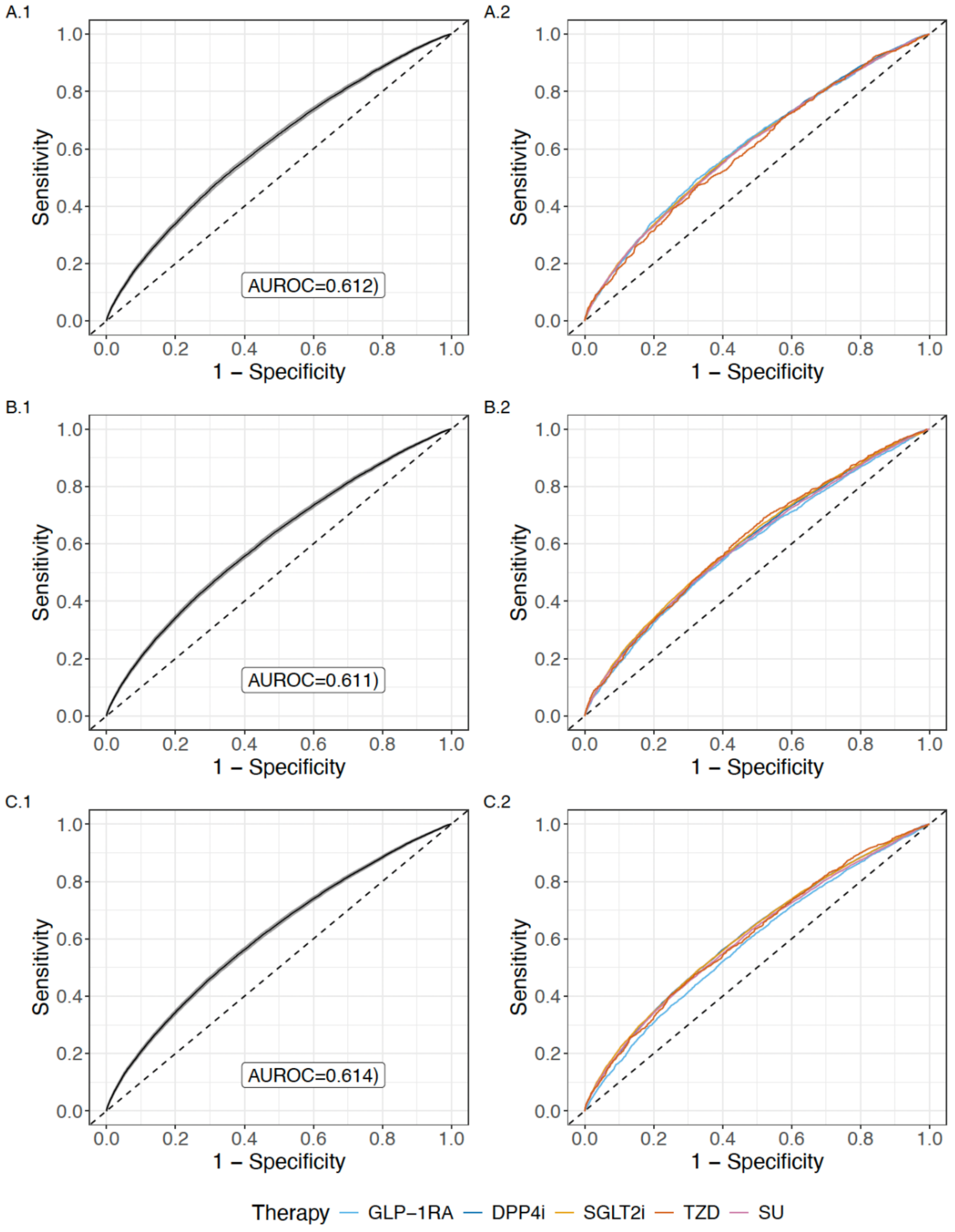

sFig. 6: Distribution of predicted CATE effects for drug pairs. Negative values reflect a predicted HbA1c treatment benefit on drug A (left side) and positive values reflect a predicted treatment benefit on drug B (right side).

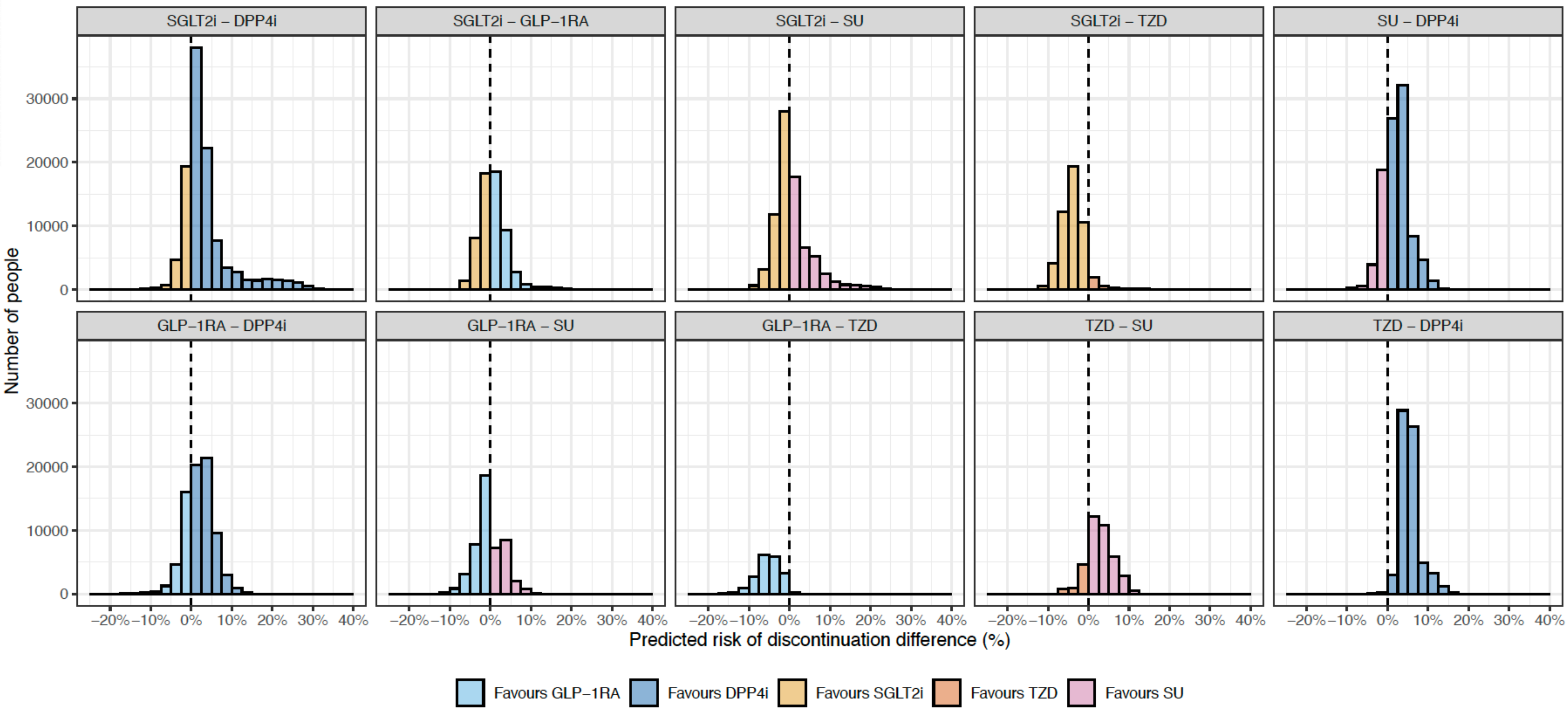

sFig. 7: Calibration of predicted heterogeneous treatment effects across drug class pairs for 3-month discontinuation. Red line represents perfect calibration. Point estimates represent predicted and observed differences in 3-month discontinuation. Error bars represent 95% Cis calculated through bootstrapping.

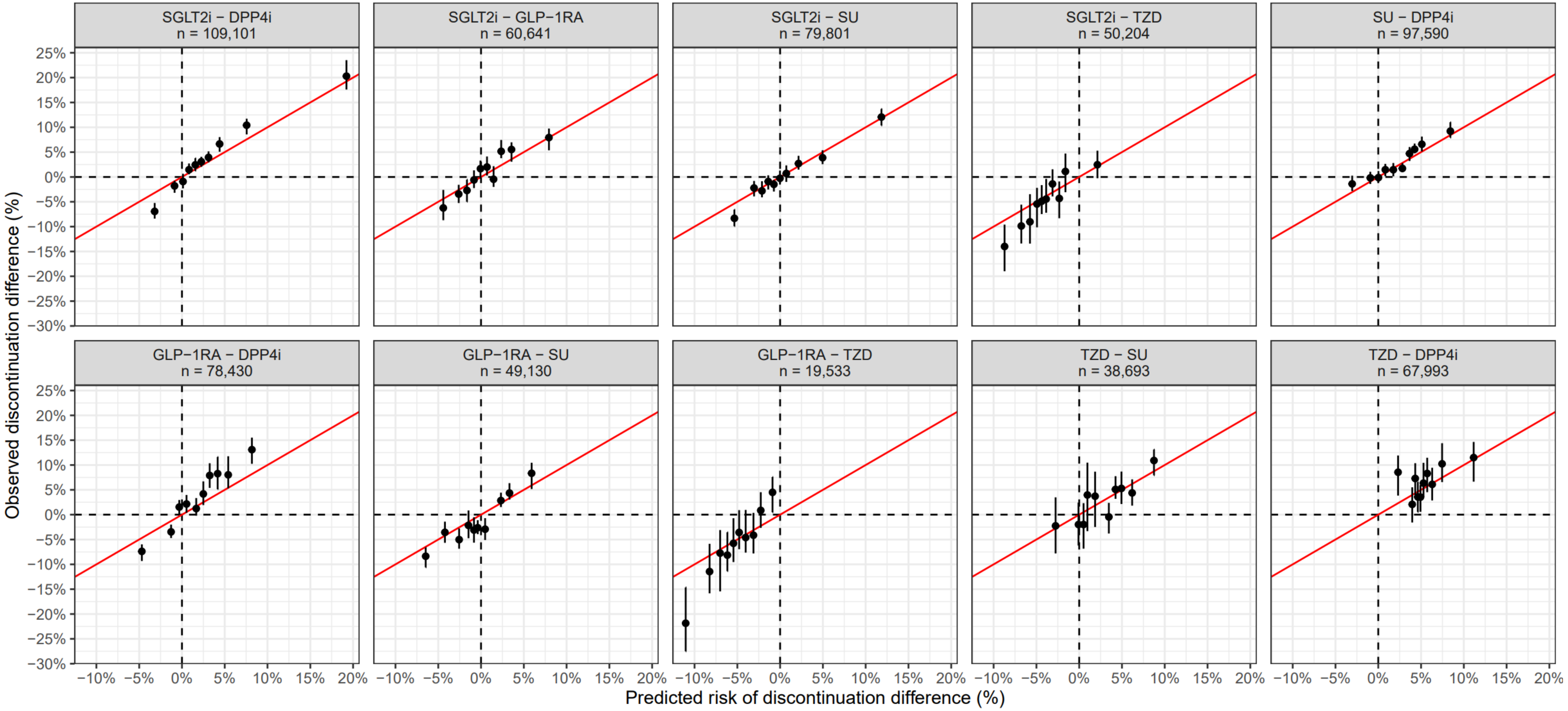

sFig. 8: Calibration of predicted heterogeneous treatment effects across drug class pairs for 6-month discontinuation. Red line represents perfect calibration. Point estimates represent predicted and observed differences in 6-month discontinuation. Error bars represent 95% Cis calculated through bootstrapping.

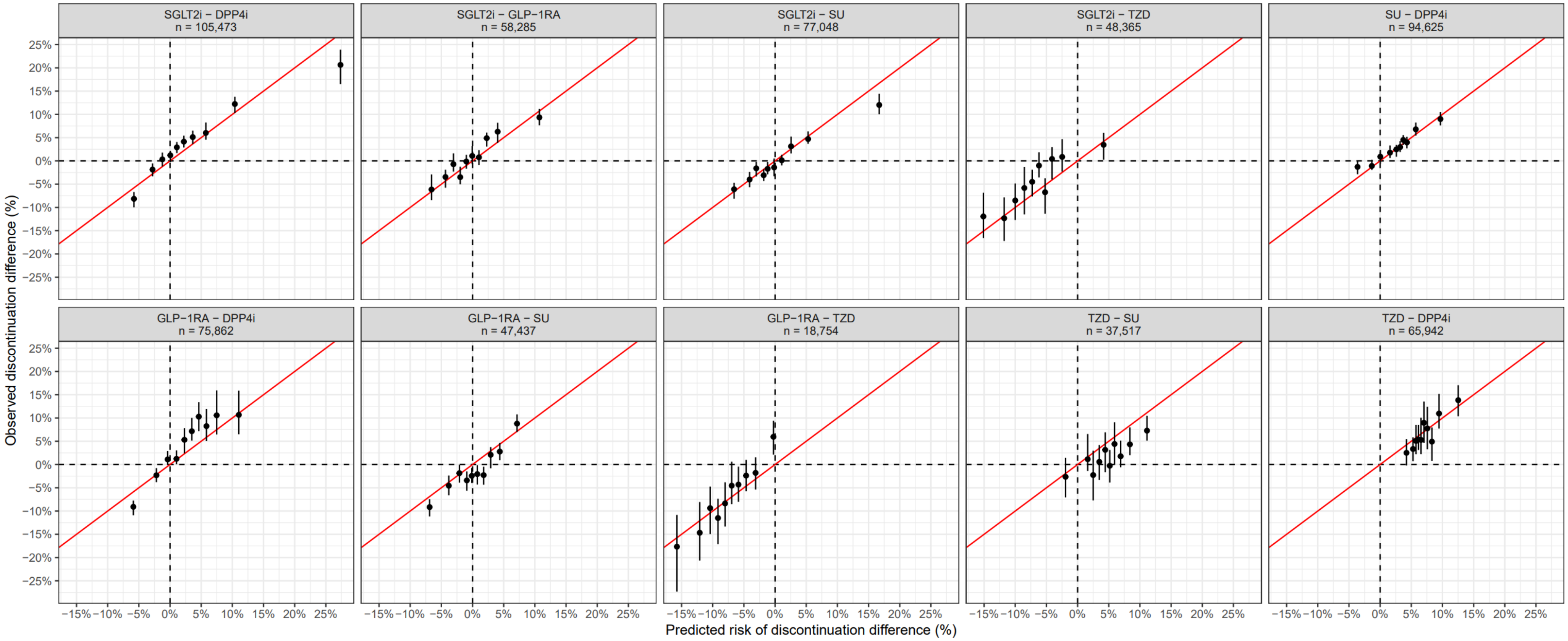

sFig. 9: Calibration of predicted heterogeneous treatment effects across drug class pairs for 12-month discontinuation. Red line represents perfect calibration. Point estimates represent predicted and observed differences in 12-month discontinuation. Error bars represent 95% Cis calculated through bootstrapping.

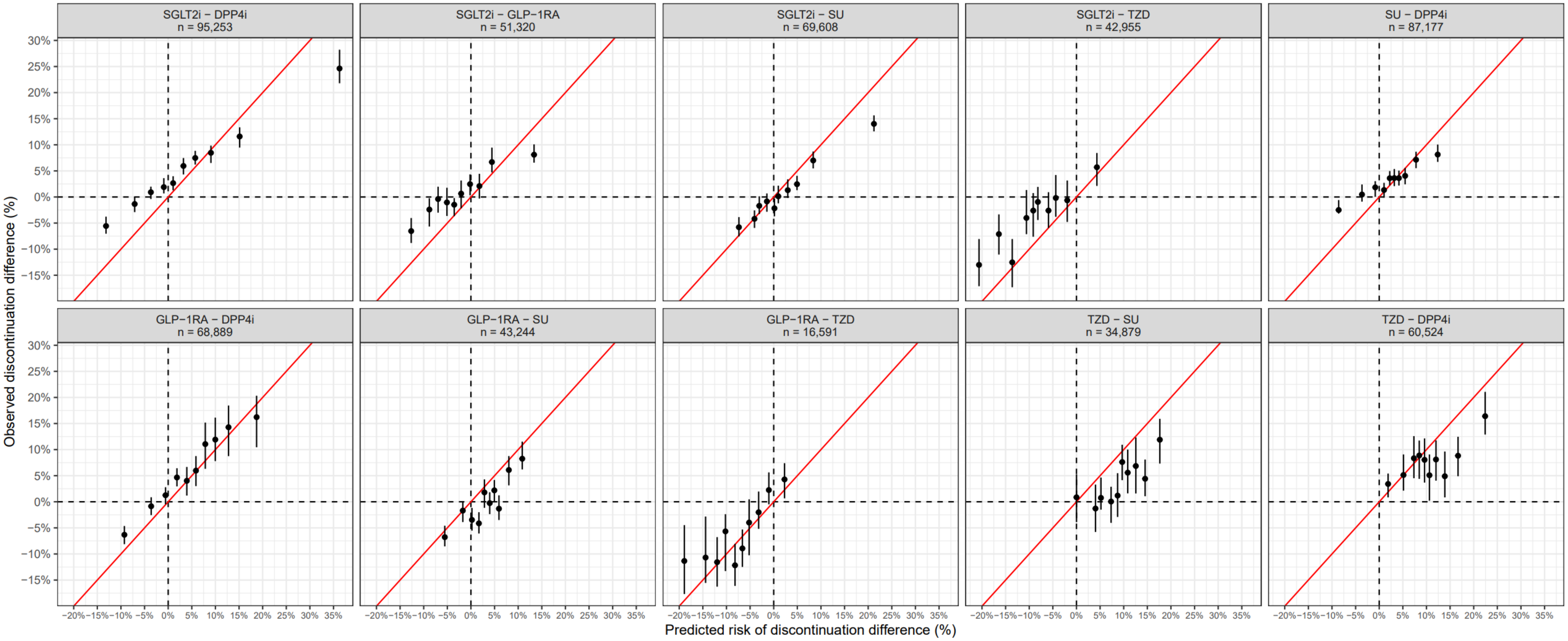

sFig. 10: Relative variable importance for 3-month discontinuation. Relative variable importance for candidate predictors demonstrating the proportion of R^2 associated with each variable in explaining the outcome.

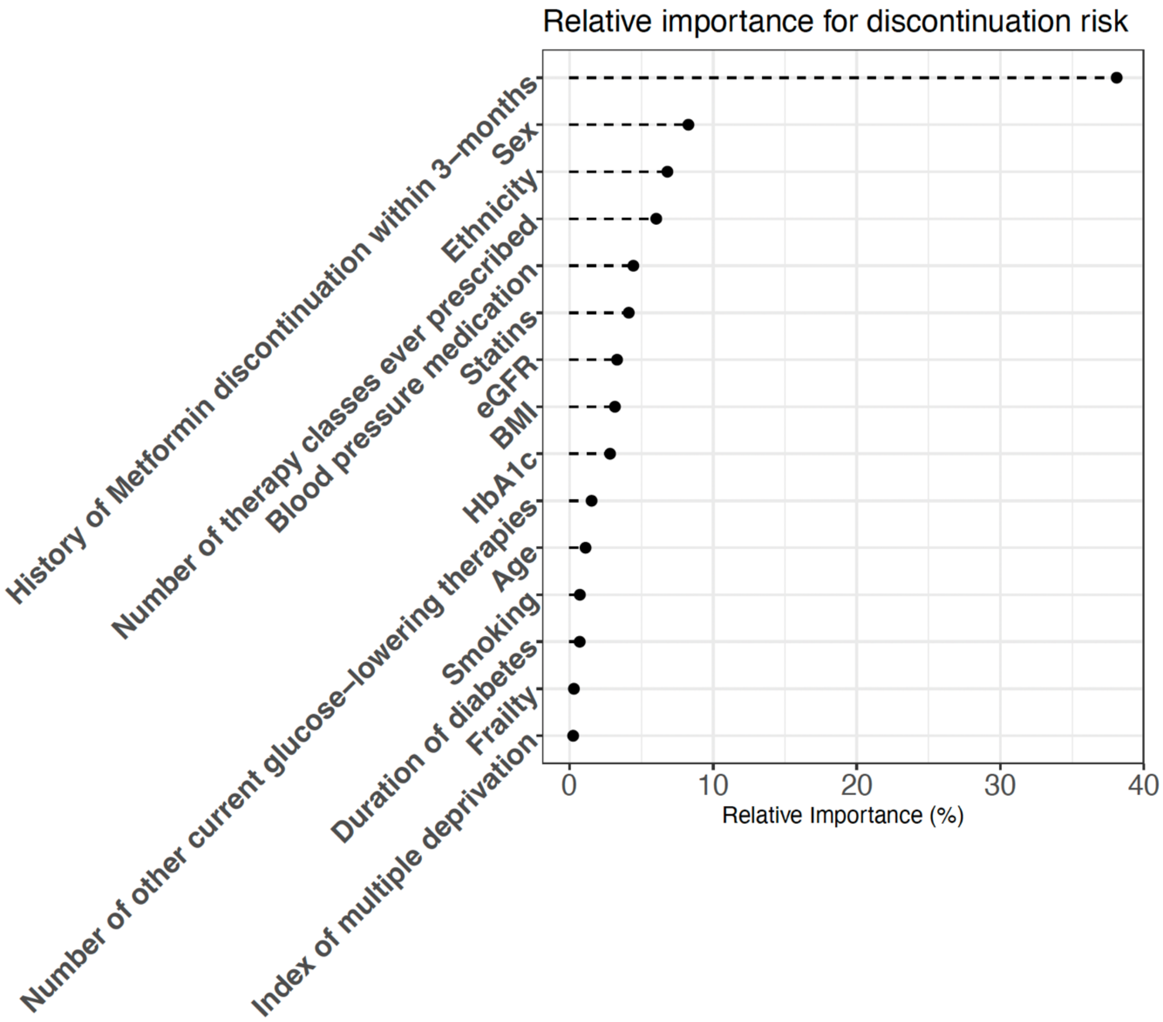

MASTERMIND consortium

Prof Andrew Hattersley[1], Prof Ewan Pearson[2], Dr Angus Jones[1], Dr Beverley Shields[1], Dr John Dennis[1], Dr Lauren Rodgers[1], Prof William Henley[1], Prof Timothy McDonald[1], Prof Michael Weedon[1], Prof Nicky Britten[1], Catherine Angwin[1], Dr Naveed Sattar[3], Dr Robert Lindsay[3], Prof Christopher Jennison[4], Prof Mark Walker[5], Prof Kennedy Cruickshank[6], Dr Salim Janmohamed[7], Prof Christopher Hyde[1], Prof Rury Holman[8], Prof Andrew Farmer[8], Prof Alastair Gray[8], Prof Stephen Gough[8], Dr Olorunsola Agbaje[8], Dr Trevelyan McKinley[1], Dr Sebastian Vollmer[9], Dr Bilal Mateen[7], Prof William Hamilton[1], Dr Katie G. Young[1], Dr Pedro Cardoso[1], Dr Laura Güdemann[1]

[1] University of Exeter
[2] University of Dundee
[3] University of Glasgow
[4] University of Bath
[5] University of Newcastle
[6] Kings College London
[7] University College London
[8] University of Oxford
[9] University of Kaiserslautern

TRIPOD+AI

Version: 11-January-2024

Section/Topic	Item	Development / evaluation[1]	Checklist item	Reported on page
**TITLE**				
Title	1	D;E	Identify the study as developing or evaluating the performance of a multivariable prediction model, the target population, and the outcome to be predicted	P.1
**ABSTRACT**				
Abstract	2	D;E	See TRIPOD+AI for Abstracts checklist	P.3-4
**INTRODUCTION**				
Background	3a	D;E	Explain the healthcare context (including whether diagnostic or prognostic) and rationale for developing or evaluating the prediction model, including references to existing models	Introduction
	3b	D;E	Describe the target population and the intended purpose of the prediction model in the context of the care pathway, including its intended users (e.g., healthcare professionals, patients, public)	Introduction
	3c	D;E	Describe any known health inequalities between sociodemographic groups	Introduction
Objectives	4	D;E	Specify the study objectives, including whether the study describes the development or validation of a prediction model (or both)	Introduction
**METHODS**				
Data	5a	D;E	Describe the sources of data separately for the development and evaluation datasets (e.g., randomised trial, cohort, routine care or registry data), the rationale for using these data, and representativeness of the data	Methods par.1
	5b	D;E	Specify the dates of the collected participant data, including start and end of participant accrual; and, if applicable, end of follow-up	Methods par.1
Participants	6a	D;E	Specify key elements of the study setting (e.g., primary care, secondary care, general population) including the number and location of centres	Methods par.1
	6b	D;E	Describe the eligibility criteria for study participants	Methods par.1
	6c	D;E	Give details of any treatments received, and how they were handled during model development or evaluation, if relevant	Methods par.1
Data preparation	7	D;E	Describe any data pre-processing and quality checking, including whether this was similar across relevant sociodemographic groups	Methods par.1
Outcome	8a	D;E	Clearly define the outcome that is being predicted and the time horizon, including how and when assessed, the rationale for choosing this outcome, and whether the method of outcome assessment is consistent across sociodemographic groups	Methods par.2
	8b	D;E	If outcome assessment requires subjective interpretation, describe the qualifications and demographic characteristics of the outcome assessors	-
	8c	D;E	Report any actions to blind assessment of the outcome to be predicted	-
Predictors	9a	D	Describe the choice of initial predictors (e.g., literature, previous models, all available predictors) and any pre-selection of predictors before model building	Methods par.3
	9b	D;E	Clearly define all predictors, including how and when they were measured (and any actions to blind assessment of predictors for the outcome and other predictors)	Methods par.3
	9c	D;E	If predictor measurement requires subjective interpretation, describe the qualifications and demographic characteristics of the predictor assessors	-
Sample size	10	D;E	Explain how the study size was arrived at (separately for development and evaluation), and justify that the study size was sufficient to answer the research question. Include details of any sample size calculation	Methods par.1
Missing data	11	D;E	Describe how missing data were handled. Provide reasons for omitting any data	Methods par.4
Analytical methods	12a	D	Describe how the data were used (e.g., for development and evaluation of model performance) in the analysis, including whether the data were partitioned, considering any sample size requirements	Methods par.4-5
	12b	D	Depending on the type of model, describe how predictors were handled in the analyses (functional form, rescaling, transformation, or any standardisation).	Methods par.4-5
	12c	D	Specify the type of model, rationale[2], all model-building steps, including any hyperparameter tuning, and method for internal validation	Methods par.5
	12d	D;E	Describe if and how any heterogeneity in estimates of model parameter values and model performance was handled and quantified across clusters (e.g., hospitals, countries). See TRIPOD-Cluster for additional considerations[3]	Methods par.5
	12e	D;E	Specify all measures and plots used (and their rationale) to evaluate model performance (e.g., discrimination, calibration, clinical utility) and, if relevant, to compare multiple models	Methods par.5
	12f	E	Describe any model updating (e.g., recalibration) arising from the model evaluation, either overall or for particular sociodemographic groups or settings	-
	12g	E	For model evaluation, describe how the model predictions were calculated (e.g., formula, code, object, application programming interface)	Methods par.5
Class imbalance	13	D;E	If class imbalance methods were used, state why and how this was done, and any subsequent methods to recalibrate the model or the model predictions	-
Fairness	14	D;E	Describe any approaches that were used to address model fairness and their rationale	-
Model output	15	D	Specify the output of the prediction model (e.g., probabilities, classification). Provide details and rationale for any classification and how the thresholds were identified	Methods par.5

[1] D=items relevant only to the development of a prediction model; E=items relating solely to the evaluation of a prediction model; D;E=items applicable to both the development and evaluation of a prediction model

[2] Separately for all model building approaches.

[3] TRIPOD-Cluster is a checklist of reporting recommendations for studies developing or validating models that explicitly account for clustering or explore heterogeneity in model performance (eg, at different hospitals or centres). Debray et al, BMJ 2023; 380: e071018 [DOI: 10.1136/bmj-2022-071018]

Training versus evaluation	16	D;E	Identify any differences between the development and evaluation data in healthcare setting, eligibility criteria, outcome, and predictors	-
Ethical approval	17	D;E	Name the institutional research board or ethics committee that approved the study and describe the participant-informed consent or the ethics committee waiver of informed consent	Methods par.1
**OPEN SCIENCE**				
Funding	18a	D;E	Give the source of funding and the role of the funders for the present study	Acknowledgements
Conflicts of interest	18b	D;E	Declare any conflicts of interest and financial disclosures for all authors	Acknowledgements
Protocol	18c	D;E	Indicate where the study protocol can be accessed or state that a protocol was not prepared	Acknowledgements
Registration	18d	D;E	Provide registration information for the study, including register name and registration number, or state that the study was not registered	Acknowledgements
Data sharing	18e	D;E	Provide details of the availability of the study data	Acknowledgements
Code sharing	18f	D;E	Provide details of the availability of the analytical code[4]	Acknowledgements
**PATIENT & PUBLIC INVOLVEMENT**				
Patient & Public Involvement	19	D;E	Provide details of any patient and public involvement during the design, conduct, reporting, interpretation, or dissemination of the study or state no involvement.	-
**RESULTS**				
Participants	20a	D;E	Describe the flow of participants through the study, including the number of participants with and without the outcome and, if applicable, a summary of the follow-up time. A diagram may be helpful.	Results par.1
	20b	D;E	Report the characteristics overall and, where applicable, for each data source or setting, including the key dates, key predictors (including demographics), treatments received, sample size, number of outcome events, follow-up time, and amount of missing data. A table may be helpful. Report any differences across key demographic groups.	Results par.1/2
	20c	E	For model evaluation, show a comparison with the development data of the distribution of important predictors (demographics, predictors, and outcome).	Results par.1/2
Model development	21	D;E	Specify the number of participants and outcome events in each analysis (e.g., for model development, hyperparameter tuning, model evaluation)	Results par.1/2
Model specification	22	D	Provide details of the full prediction model (e.g., formula, code, object, application programming interface) to allow predictions in new individuals and to enable third-party evaluation and implementation, including any restrictions to access or re-use (e.g., freely available, proprietary)[5]	Results par.3
Model performance	23a	D;E	Report model performance estimates with confidence intervals, including for any key subgroups (e.g., sociodemographic). Consider plots to aid presentation.	Results par.3/4
	23b	D;E	If examined, report results of any heterogeneity in model performance across clusters. See TRIPOD Cluster for additional details[3].	-
Model updating	24	E	Report the results from any model updating, including the updated model and subsequent performance	-
**DISCUSSION**				
Interpretation	25	D;E	Give an overall interpretation of the main results, including issues of fairness in the context of the objectives and previous studies	Discussion par.1/2
Limitations	26	D;E	Discuss any limitations of the study (such as a non-representative sample, sample size, overfitting, missing data) and their effects on any biases, statistical uncertainty, and generalizability	Discussion par.3
Usability of the model in the context of current care	27a	D	Describe how poor quality or unavailable input data (e.g., predictor values) should be assessed and handled when implementing the prediction model	-
	27b	D	Specify whether users will be required to interact in the handling of the input data or use of the model, and what level of expertise is required of users	-
	27c	D;E	Discuss any next steps for future research, with a specific view to applicability and generalizability of the model	Discussion par.4

From: Collins GS, Moons KGM, Dhiman P, et al. *BMJ* 2024;385:e078378. doi:10.1136/bmj-2023-078378

[4] This relates to the analysis code, for example, any data cleaning, feature engineering, model building, evaluation.
[5] This relates to the code to implement the model to get estimates of risk for a new individual.
